# Prenylated *Trans*-Cinnamic Esters and Ethers against Clinical *Fusarium* spp.: Repositioning of Natural Compounds in Antimicrobial Discovery

**DOI:** 10.3390/molecules26030658

**Published:** 2021-01-27

**Authors:** Safa Oufensou, Stefano Casalini, Virgilio Balmas, Paola Carta, Wiem Chtioui, Maria A. Dettori, Davide Fabbri, Quirico Migheli, Giovanna Delogu

**Affiliations:** 1 Dipartimento di Agraria, Università degli Studi di Sassari, Via E. De Nicola 9, 07100 Sassari, Italy; soufensou@uniss.it (S.O.); stefanocasalini66@yahoo.it (S.C.); balmas@uniss.it (V.B.); w.chtioui@studenti.uniss.it (W.C.); qmigheli@uniss.it (Q.M.); 2 Istituto CNR di Chimica Biomolecolare, Traversa La Crucca 3, 07100 Sassari, Italy; paola.carta@cnr.it (P.C.); davidegaetano.fabbri@cnr.it (D.F.); giovanna.delogu@icb.cnr.it (G.D.); 3 Nucleo di Ricerca sulla Desertificazione, Università degli Studi di Sassari, Via E. De Nicola 9, 07100 Sassari, Italy

**Keywords:** onychomycosis, mycoses, *Fusarium* spp., drug development, antifungal activity, phenolic inhibitors, hydroxycinnamic acid derivates, *p*-coumaric acid 3,3′-dimethyl allyl ester

## Abstract

Onychomycosis is a common nail infection mainly caused by species belonging to the *F. oxysporum*, *F. solani*, and *F. fujikuroi* species complexes. The aim of this study was to evaluate the in vitro susceptibility of six representative strains of clinically relevant *Fusarium* spp. toward a set of natural-occurring hydroxycinnamic acids and their derivatives with the purpose to develop naturally occurring products in order to cope with emerging resistance phenomena. By introducing a prenylated chain at one of the hydroxy groups of *trans*-cinnamic acids **1**–**3**, ten prenylated derivatives (coded **4**–**13**) were preliminarily investigated in solid Fusarium minimal medium (FMM). Minimal inhibitory concentration (MIC) and lethal dose 50 (LD_50_) values were then determined in liquid FMM for the most active selected antifungal *p*-coumaric acid 3,3′-dimethyl allyl ester **13**, in comparison with the conventional fungicides terbinafine (TRB) and amphotericin B (AmB), through the quantification of the fungal growth. Significant growth inhibition was observed for prenylated derivatives **4**–**13**, evidencing ester **13** as the most active. This compound presented MIC and LD_50_ values (62–250 µM and 7.8–125 µM, respectively) comparable to those determined for TRB and AmB in the majority of the tested pathogenic strains. The position and size of the prenylated chain and the presence of a free phenol OH group appear crucial for the antifungal activity. This work represents the first report on the activity of prenylated cinnamic esters and ethers against clinical *Fusarium* spp. and opens new avenues in the development of alternative antifungal compounds based on a drug repositioning strategy.

## 1. Introduction

**Figure 1:**
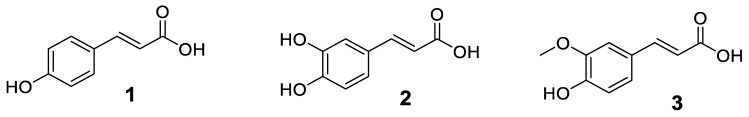
Chemical structures of *p*-coumaric acid **1**, caffeic acid **2**, and ferulic acid **3**.

Phenolics are among the most desirable food components because of their excellent antioxidant activity and nutraceutical properties. They also find wide-ranging application in medicine and agriculture in virtue of their antimicrobial, anti-inflammatory, and antitumoral activities [1,2]. Among the phenolic compounds, cinnamic acids are a group of aromatic carboxylic acids formed through the biochemical route of shikimate pathway, leading to the synthesis of lignin, the polymeric material that provides mechanical support to the plant cell wall [3,4]. *p*-Coumaric acid **1**, caffeic acid **2**, and ferulic acid **3** are the most common cinnamic acids, consisting of a *trans*-α, β-unsaturated carboxylic chain bonded to a phenol, catechol, and guaiacyl unit, respectively. They possess three distinctive structural motifs that may possibly contribute to the free radical scavenging capability of these compounds (Figure 1).

The presence of an electron donating group on the benzene rings provides the additional property of terminating free radical chain reaction. The carboxylic acid group with a conjugated C-C double bond provides additional quenching sites for free radicals. These features of cinnamic acids are reflected in many biological processes. Cinnamic acids, the main constituents of plant defense, prevent the effects of reactive oxygen species (ROS) formed during fungal infection [5]. The carboxylic acid group of cinnamic acid can act as an anchor by which the compound binds to the lipid bilayer, providing some protection against lipid peroxidation, a process spread out across mammal and human cells [6].

Cinnamic acids **1**–**3** are widely distributed in fruits (e.g., apple, pear, grape, orange, tomato, and berries), vegetables (e.g., bean, potato, and onion), and cereals (e.g., maize, oat, and wheat bran) [7]. They occupy a key place as intermediates in the synthesis of pharmaceuticals, dyes, flavorings, cosmetics, thermoplastics, and materials [8,9]. Because of their high-promoting health capacities and commercial value and given the availability of cinnamic acids **1**–**3** in different plants, extracting processes from biomass are also well studied even though the main accessibility of these compounds comes from synthetic or microbial processes [10,11].

Drug repositioning implies the identification of novel biological targets for natural-occurring compounds that may find application in fields where safety and efficiency are still lacking. One approach relies on slight structural modification of the natural compound, aiming to enhance its biological activity toward a specific target. When the structural modification of the compound concerns the introduction of a natural unit to the molecule framework, the final compound acquires a natural-like feature. Often, such slight structural modifications are devoted to improving bioavailability and selectivity of the parent compound [12,13,14,15,16].

Onychomycosis is a chronic fungal nail infection mainly caused by *Fusarium* spp., particularly those belonging to three specie complexes: *F. oxysporum* (FOSC), *F. solani* (FSSC), and *F. fujikuroi* species complex (FFSC) [17,18,19,20,21]. *F. oxysporum* Schlechtend. emend. Snyder & Hansen; *F. solani* (Mart.) Sacc.; *F. petroliphilum* (Q.T. Chen & X.H. Fu) Geiser, O’Donnell, D.P.G. Short, & N. Zhang; *F. keratoplasticum* Geiser, O’Donnell, D.P.G. Short, & Ning Zhang; *F. falciforme* (Carrión); and *F. verticillioides* (Saccardo) Nirenberg are the most representative species responsible for onychomycosis; moreover, they also infect skin and hair and are considered as emerging pathogens from superficial mycoses as dermatomycoses. *Fusarium* spp. are increasingly reported among the world population. Besides dermatomycoses or onycomycoses, they are responsible for disseminated infections, particularly in patients undergoing cancer therapy or those affected by immunological deficiency [22,23,24].

Systemic antifungals are the most effective treatment, with meta-analyses showing mycotic cure rates of 76% for terbinafine, 63% for itraconazole with pulse dosing, 59% for itraconazole with continuous dosing, and 48% for fluconazole [25,26]. The use of these agents is discouraged in patients suffering from liver, renal, or heart disease, and in those receiving medications with which there may be significant drug–drug interactions [27]. Terbinafine (TRB) belongs to the allylamine class of synthetic antimycotic agents, and inhibits the squalene epoxidase, a key enzyme involved in the early phase of the ergosterol biosynthetic pathway [28]. Amphotericin B (AmB) is a broad-spectrum antifungal agent belonging to the polyene class; its mechanism of action targets membrane function by forming channels in the fungal cell membrane, hence allowing ions and organic compounds of the cytoplasm to escape [29].

Susceptibility to fungicides among different *Fusarium* spp. may vary greatly, and clinical *Fusaria* showing multiple resistance to most applied antifungal drugs are increasingly being reported [30,31,32]. Numerous factors have been cited to explain the lack of response to therapy, such as nonadherence to treatment, incorrect diagnosis, or advanced disease [25]. Additionally, antimycotic prophylaxis in high-risk patients may enhance selective pressure, which favors multidrug-resistant fungi, including *Fusaria* [33]. This urges a massive investment in the development of novel agents to treat emerging and resistant fungi [34].

Considering the antimicrobial activity of cinnamic acids **1**–**3** [35], their low toxicity, and large market availability, the aim of this work was to investigate these compounds against a selection of representative strains belonging to the *F. oxysporum* (FOSC), *F. solani* (FSSC), and *F. fujikuroi* (FFSC) species complexes; namely, *F. oxysporum*, *F. solani F. keratoplasticum*, and *F. verticillioides*.

**Figure 2:**
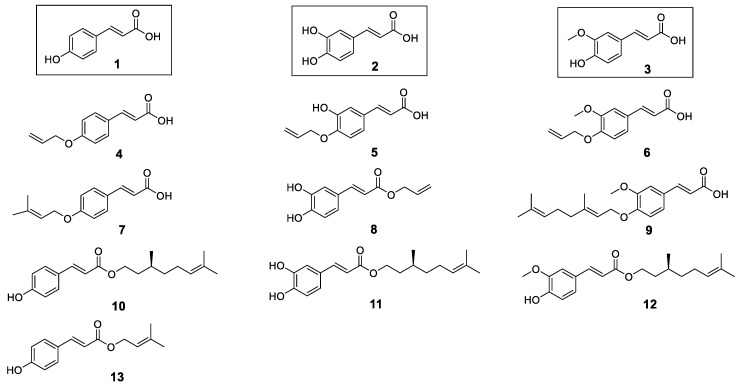
Chemical structure of the tested compounds numbered from **1** to **13**.

Aiming to improve the bioavailability and to enhance antimicrobial activity, we transformed cinnamic acids **1**–**3** in esters and ethers using short, medium, and long prenylated chains as alcoholic unit. The efficiency of prenylated phenols in crossing bacterial and fungal membranes [36], as well as their role in exerting antimicrobial activity [37], are generally acknowledged. *O*-prenylated phenols are secondary metabolites of plants. Even though they have been considered for years as biosynthetic intermediates of the most widespread *C*-prenylated derivatives, recently, *O*-prenylated chains are assuming a key role in the bioactivity of molecules into which they are embedded [38,39]. We thus predicted that the preparation of prenylated ethers and prenylated esters of cinnamic acids **1**–**3** (Figure 2) could offer an alternative to the conventional antifungal drugs used against clinical *Fusaria*, enhancing the antimycotic effect of the parent acid.

An in vitro assay of clinical isolates of *Fusarium* spp. grown on solid Fusarium minimal medium (FMM) amended with compounds **1**–**13** was carried out and a structure–activity relationship was described. Minimal inhibitory concentration (MIC) and lethal dose 50 (LD_50_) values were then determined in liquid FMM for the most active selected antifungal compound, in comparison with the conventional fungicides terbinafine (TRB) and amphotericin B (AmB). The presence of a *O*-prenylated chain in natural occurring cinnamic acids may share a different mode of action compared to conventional antifungal drugs and offers a successful example of drug repositioning.

## 2. Results

### 2.1. Chemistry

Prenylated esters **4**–**7** and **9** and prenylated ethers **10**–**13** were prepared, starting from the corresponding cinnamic acid **1**–**3** and, according to the reaction, allyl, 3,3′-dimethyl allyl (prenyl), geranyl, and citronellyl bromide or alcohol. Because of the contemporary presence of two hydroxyl groups in the starting hydroxy cinnamic acid, different synthetic approaches were applied when ether or ester was the final product.

**Scheme 1:**
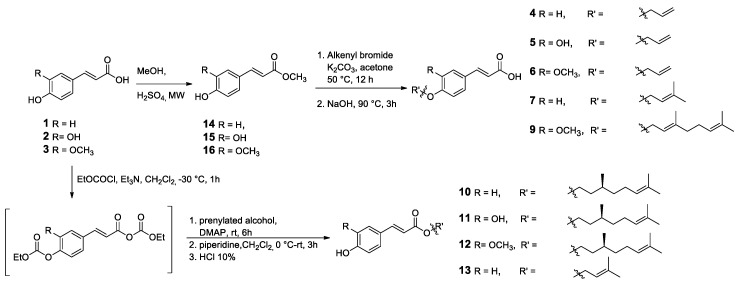
Synthesis of hydroxyl cinnamic ethers **4**–**7** and **9** and esters **10**–**13** and **14**–**16**.

In order to functionalize selectively the phenolic-OH group with a prenylated chain, esterification of the carboxylic group was mandatory (Scheme 1).

Ethers **4**–**7** and **9** were obtained by the Williamson reaction, starting from the corresponding methyl ester and the appropriate alkenyl bromide under basic conditions and further ester hydrolysis. Yields varied in the range of 53 and 85%. Methyl esterification of acids **1**–**3** was carried out under acid conditions under microwave treatment.

For compound 5, the selectivity of etherification reaction at the *p*-phenolic-OH was confirmed by Nuclear Overhauser Effect Spectroscopy-NMR (NOESY) experiment and by comparison of NMR spectra with the corresponding methyl ester reported in the literature (see Materials and Methods for references).

Esters **10**–**13** were prepared with yields ranging between 37 and 47%, starting from the corresponding parent acid (i.e., 4-hydroxy cinnamic acid **1**, caffeic acid **2**, and ferulic acid **3**) via activation of the carboxylic group with ethyl chloroformate and triethylamine and further addition of the appropriate prenylated alcohol (Scheme 1) and hydrolysis of the phenyl ethyl carbonate.

Compound **8** was obtained via Fischer esterification of acid **2** using allyl alcohol as solvent under acidic conditions as reported in the literature. Compound **13** was also obtained in 87% yield by enzymatic transesterification of the corresponding ethyl ester, in turn achieved by the microwave method, as well as 3,3′-dimethyl allyl alcohol, in mild conditions.

The purity of all new compounds was judged to be >98% by ^1^H-NMR spectral determination.

The remarkable different lipophilicity estimated, as the value of the logarithm of the partition coefficient of compounds **1**–**13** for *n*-octanol/water (LogP), allowed us to evaluate the possible influence of this property in the antifungal activity of each compound (Table 1).

As expected, in all compounds studied, the lipophilicity increases as the number of carbon atoms in the prenylated chain increases. Among the three natural occurring acids, the lipophilicity changes in the following order: ferulic acid **3** > caffeic acid **2** > *p*-coumaric acid **1**. The trend is also retained both in the series of geranyl esters **10** > **12** > **11** and in the series of allyl ethers **4** > **6** > **5**. No difference in LogP value resulted in caffeic acid derivatives when the *p*-phenolic-OH or the carboxylic functionalities were protected by an allyl group (i.e., compounds **5** and **8**) or by a 3,3′-dimethyl allyl chain (i.e., compounds **7** and **13**).

### 2.2. Antifungal Activity of the Parent Compounds ***1**–**3*** and Derivatives

The three naturally occurring hydroxycinnamic acids **1**–**3** and their ethers and esters derivatives **4**–**13** (Figure 2) were used in a preliminary in vitro screening to compare their antifungal activity against six *Fusarium* spp. clinical isolates associated to onychomycosis in a hospital in Milan (Italy) (Table 2).

In the first screening, carried out on solid FMM, exposure to cinnamic acids **1–3** did not determine any significant reduction of mycelium fungal growth compared with the untreated control, with the exception of caffeic acid **2**, which induced a slight inhibition on *F. oxysporum* 89 colony diameter (Figure S1).

In the case of FOSC and FFSC, we noted a significant inhibition of vegetative growth upon exposure to all the compounds derived from cinnamic acids **1**–**3** compared with the untreated control, except for compound **5**, which did not induce any significant reduction of colony diameter in the two FOSC isolates. FOSC isolates were particularly sensitive (>53% and >56% of inhibition for *F. oxysporum* 89 and *F. oxysporum* 91, respectively) to compounds **4**, **7**, **8**, **9**, and **13**, whereas in the case of FFSC, the most effective compounds were **5**, **8**, **9**, **10**, **11**, and **13**. In both species complexes, compound **13** was by far the most effective inhibitor of fungal growth, leading to complete inhibition of the two *F. verticillioides* isolates (Figures S1–S3).

The two representative isolates of the FSSC displayed a different level of sensitivity to compounds **4**–**13**: compounds **4**, **7**, **10**, **11**, and **13** determined >25% inhibition of radial growth on *F. keratoplasticum* 93, whose vegetative growth on solid FMM was completely inhibited by compound **13**, whereas *F. solani* 96 was only partially inhibited by compounds **10**, **11** (36–30% inhibition, respectively), and **13** (63% inhibition; Figure S2).

These preliminary data demonstrate unequivocally that compound **13** has the highest antifungal activity towards all *Fusarium* strains investigated (Figures S1–S3). Citronellyl *p*-coumaric ester **10** was the second most effective inhibitor towards FSSC and FFSC strains, while FOSC representative isolates proved more sensitive to compound **8**. Compound **9** was more effective on FOSC and FFSC representatives compared with FSSC ones. The inhibitory activity of compound **9** towards FOSC isolates was comparable to that displayed by the ethers **4** and **7**, presenting a shorter prenylated chain. Overall, the ester functionality in the cinnamic acid derivatives appears more efficient in conferring inhibitory properties to the tested compounds compared with the ether one.

### 2.3. Determination of the Minimal Inhibitory Concentration (MIC) and Lethal Dose 50 (LD_50_) for p-Coumaric acid 3,3′-Dimethyl Allyl Ester ***13***

In a further screening performed on FMM liquid medium, the minimal inhibitory concentration (MIC) and the lethal dose 50 (LD_50_) of compound **13** and of two conventional fungicides were determined (Table 3). *p*-Coumaric acid 3,3′-dimethyl allyl ester **13** confirmed its remarkable good antifungal activity already shown in solid FMM compared with TRB and AmB, with an MIC range comprised between 125 and 250 µM in FOSC (Figures S4 and S5), 62 and 125 µM in FSSC (Figures S6 and S7), and 125 and 500 µM in FFSC (Figures S8 and S9) representative isolates. With respect to the LD_50_, the most effective compound was TRB (LD_50_ ranging from 2.0 to 64 µM), followed by AmB (LD_50_ ranging from 1.0 to 67.5 µM) and ester **13** (LD_50_ ranging from <7.8 to 125 µM), respectively, for almost all strains. In the case of *F. verticillioides* 115, which was less sensitive to AmB (LD_50_ 8.40–16.8 µM) than to TRB (LD_50_ 2.0–4.0 µM) and **13**, the latter was able to reduce 50% of the fungal growth at a concentration <7.8 µM (Table 3, Figure S9).

The effects of compounds **13**, TRB, and AmB applied at the same concentration as the MIC determined in liquid FMM on the morphology of representative isolates of *F. solani, F. keratoplasticum, F. oxysporum*, and *F. verticillioides* were examined by optical microscopy after 72 h (Table 4). While in the untreated controls, a regular morphology of the mycelium with abundant presence of microconidia as well as normal spore germination were observed, in the presence of 250 μM of compound **13**, degradation of the hyphae and vacuolisation of the cytoplasm were evident, along with an alteration of the rarely formed conidia, showing a compromised germination. The release of cell constituents was also noticeable, which can explain the progressive increase in the absorbance signal over time in liquid FMM (Figures S4–S9).

After 72 h of exposure to 256 µM TRB, a marked reduction on mycelium formation was observed, and fungal hyphae and spores showed a distorted morphology with tortuous growth (Table 4). This concentration of TRB could not totally inhibit the mycelium growth or the spore production but caused an evident swelling of conidia. The effects of TRB were more noticeable compared with those induced by AmB at the MIC of 135 µM, causing only some mycelial distortion and fewer microconidia compared with the control (Table 4).

## 3. Discussion

By introduction of a short, medium, or long prenylated chain at one of the hydroxyl functionalities of *trans*-cinnamic acids **1**–**3**, a series of prenylated cinnamic derivatives **4**–**13** was prepared to test the hypothesis that differences in the chemical and physical proprieties would influence the antifungal activity of the compounds against clinical *Fusarium* spp. The presence of a conjugated double bond confers particular conformational and electronic characteristic to these compounds strongly influenced by the phenol-OH group in para position. In order to observe the effect of the hydroxylated aromatic ring on the fungicide activity, a set of cinnamic esters **10**–**12** was prepared by functionalisation of the corresponding carboxylic acid with citronellol, while a set of allyl cinnamic ethers **4**–**6** was prepared with the aim to evaluate the influence of the phenolic-OH group in para position to the alpha, beta-unsaturated carboxylic chain. No significant differences were observed in the synthesis and yields of each set of compounds with different aromatic rings, whereas lower yields in esters in comparison with ethers were achieved, evidencing the higher reactivity of the phenolic-OH group.

The Food and Drug Administration (FDA) considers *trans*-cinnamic acids **1**–**3** as “generally recognized as safe” (GRAS), enabling their use in the field of food additives [40]. Compounds **1**–**3** are commercially available at an affordable price and can been obtained by direct extraction from plants biomass where the compounds are the main components [41] or by chemical and biotechnological processes [42]. Besides cinnamic acids **1**–**3**, prenylated cinnamic ester **13** and ethers **7** and **9** are plant components, whose extracts were studied for their remarkable biological properties [43]. In particular, compounds **9** and **13** are present in propolis, a source of valuable compounds with antioxidant and antimicrobial activity [44]. Compound **9** is not toxic to human cells and presents antitumoral and anti-inflammatory activities, in addition to acting as an inhibitor of biofilm formation by oral pathogenic bacteria [45]. While 4′-geranyloxy ferulic acid **9** is generally extracted from citrus fruit, quinoa seeds, and several vegetable oils, compound **13** was only detected in propolis extract. Propolis, produced by honeybees, is a very complex mixture composed of 50% resin, 30% wax, 10% essential oil, and 5% of polyphenols as flavonoids, terpenes, fatty acids, stilbenes, β-steroids, cinnamic acids, and their prenylated derivatives [46,47]. Change in chemical composition of propolis is frequently observed [44]. A few studies have been conducted on the antifungal and antibiofilm activity of propolis against onychomycosis caused by *Fusarium* spp. [48,49], but no studies aimed to identify the active component of the propolis extract against these fungi.

In a previous article reporting the activity of natural phenols against clinical *Fusarium* spp., we observed that the percentage of growth inhibition measured in liquid medium (Vogel’s) and solid (PDA) was comparable [50]. To achieve full solubilisation of compounds **1**–**13** at 0.5 mM, the preliminary screening was carried out with a sustainable solid medium based on gellan/water, where each compound was solubilized in a 0.1% water/gellan solution. In the preliminary in vitro screening of compounds **1**–**13** against *Fusarium* spp., cinnamic acids **1**–**3** were generally ineffective, whereas significant growth inhibition was achieved by prenylated derivatives **4**–**13**, evidencing ester **13** as the most active. Among the *Fusarium* spp. investigated, *F. solani* was the most resistant to compounds **1**–**13**, whereas *F. verticillioides* was the most sensitive, in accordance with data present in the literature for these species. In fact, *Fusarium* spp. are increasingly reported as resistant to many antifungal compounds in vitro; among them, *F. solani* is considered as the most resistant taxon, albeit some reports pointed out that the resistance may be species- and even isolate-dependent [51].

The antifungal activity of compounds **1**–**13** may be explained by the key role played by some moieties of their structure. The prenylated chain present in compounds **4**–**13** has the ability to penetrate and to accumulate within the fungal cell membrane, resulting, according to the size, in the disruption of its integrity as generally acknowledged for prenylated phenols [36,37,38]. The position and size of the prenylated chain in the studied compounds appear crucial for their antifungal activity. Although we did not perform a proper bioavailability assay, we observed a detrimental effect on the fungal membrane when treated with compound **13** (Table 4).

Esters were more active than ethers as inhibitors of all tested *Fusarium* spp. Ester **13** was definitely more active than the corresponding ether **7** containing the same 3,3′-dimethyl allyl moiety, even though an identical lipophilicity was measured for both compounds (LogP 3.04). In the esters series, the alcoholic unit represented by a 3,3′-dimethyl allyl chain (compound **13**) was more active than the citronellyl one (compound **10**), a substituent that significantly increases the lipophilicity of the molecule (LogP 4.9).

We suppose that different prenylated chains may change the bioavailability of the compound influencing the hydrolytic degradation of the prenylated esters within the fungal cell. In fact, hydrolytic degradation, mediated by fungal enzymes, of the esters in the parent cinnamic acid and the corresponding alcohol cannot be ruled out. In esters **11** and **10**, a too long prenylated chain could be partially metabolized by the fungus at the first stages of contact, whereas in ester **13**, the hydrolysis would take a longer time, allowing it to reach sensitive compartments of the fungal cell where the prenyl alcohol may exert its antifungal activity. A similar effect has been reported by farnesol on *F. keratoplasticum*, which is associated with biofilm formation in hospital water systems and internal pipelines: this prenylated alcohol has a remarkable anti-biofilm activity; causes the destruction of hyphae and of the extracellular matrix; and prevents the adhesion of conidia, filamentation, and the formation of biofilm [52].

Compounds **4**–**13** contain an α,β-unsaturated Michael acceptor pharmacophore effective in interacting with nucleophiles present in the fungal cell; nevertheless, this feature is not exhaustive for the antifungal activity. The presence of a free phenol-OH in para position would play a key role in the radical scavenging and stabilisation of the radical by electronic delocalisation along the structure. In general, we observed that compounds with a catechol and guaiacyl ring favouring an intramolecular hydrogen bond and hampering the availability of the H donor to scavenge radicals were less active as antimycotic (compounds **5**, **8**, and **12**).

The antifungal activity of compound **13** was compared with that of TRB and AmB, two of the most effective conventional fungicides for clinical use [53]. TRB and AmB were applied at clinical dosage ranging between 2–256 µM and 1–135 µM, respectively, whereas compounds **13** was amended at concentrations comprised between 7.8 and 500 µM. Both TRB and AmB interact at the level of fungal cell membrane, the first one by inhibiting squalene epoxidase, a key step along the ergosterol biosynthesis pathway, and the second one by a complex interaction with phospholipid bilayers [54]. Our results demonstrate that compound **13** presents MIC and LD_50_ values against *F. verticillioides* 115 and *F. oxysporum* 89 that are consistent with those reported for AmB. Similarly, while AmB was indeed the most effective compound in terms of MIC and LD_50_ against *F. keratoplasticum* 93 and *F. solani* 96, the antifungal efficacy of compound **13** against these members of the FSSC was comparable to that of terbinafine. Besides its remarkable biological activity, ester **13** presents some attractive advantages; that is, it is as natural compound with a simple structure, a straightforward synthesis, low production cost with easy recovery of the starting materials. Considering the increasing frequency of multi-drug resistance patterns in opportunistic *Fusarium* spp. [55], the development of compound **13** as an effective antifungal drug represents a valuable alternative to the conventional therapeutic agents in onychomycosis treatment.

The results of this study provide useful insights to the optimal design of the structure of cinnamic esters with improved antifungal properties. Although cinnamic acids and their derivatives have been studied on some plant pathogenic fungi [56], to the best of our knowledge, no investigation was conducted on prenylated cinnamic esters and ethers on clinical *Fusarium* spp., thereby offering an intriguing opportunity in drug repositioning strategy.

## 4. Materials and Methods

### 4.1. Chemical Synthesis

#### 4.1.1. General

Unless otherwise noted, starting materials and reagents were obtained from commercial suppliers and were used without further purification. Melting points were determined on a Büchi 530 apparatus and are uncorrected. All ^1^H-NMR and ^13^C-NMR spectra were recorded in CDCl_3_ (if not otherwise indicated) solution at 399.94 MHz and 75.42 MHz, respectively, with a Varian VXR 5000 spectrometer (Varian, Palo Alto, CA USA). Chemical shifts are given in ppm (δ); multiplicities are indicated by s (singlet), d (doublet), t (triplet), q (quartet), m (multiplet), or dd (doublet of doublets). Elemental analyses were performed using an elemental analyser model 240 C (Perkin-Elmer, Walthan, MA USA). Acetone was freshly distilled from CaCl_2_. Flash chromatography was carried out with silica gel 60 (230–400 mesh; VWR; Radnor, AF, USA) eluting with appropriate solution in the stated v:v proportions. Analytical thin-layer chromatography (TLC) was performed with 0.25 mm thick silica gel plates (Sigma Aldrich, Munich, Germany). All reactions were monitored by TLC performed on 0.2 mm thick silica gel plates (60 F254 Merck). Microwave reactions were carried out on a MW instrument (CEM-Discover SP MW, Matthews, NC, USA). Melting points were determined on a 530 apparatus (Büchi, Flawil, Switzerland) and are uncorrected. The purity of all new compounds was judged to be >98% by ^1^H-NMR spectral determination. Lipase from *Candida antarctica* (Novozym 435 CAL-B) is immobilized on a macroporous polyacrylic resin beads (recombinant, expressed in *Aspergillus niger*, activity ≥ 5000 PLU/g (propyl laurate units/g) and purchased from Merck (Milan, Italy). Compound **8** was prepared according to the literature [57].

Lipophilicity of the compounds was estimated using the logarithm of the partition coefficient for *n*-octanol/water (log P), which was calculated using 403 ChemBioDraw Ultra 13.0.

#### 4.1.2. General Procedure for the Synthesis of Compounds **10**–**13**

Ethyl chloroformate (2 eq for **10**, **12**, and **13** or 3 eq for **11**) and triethylamine (2 eq for **10**, **12**, and **13** or 3 eq for **11**) were added to a suspension of appropriate cinnamic acid (1 eq) in dichloromethane (10 mL) and stirred for 1 h at −30 °C until all of the starting material disappeared, as determined by TLC. Appropriate alcohol (1 eq) and 4-dimethylaminopyridine (0.2 eq) were then added, and the mixture was stirred at room temperature for 6 h. The reaction mixture was acidified with hydrochloridric acid (10% solution) and extracted with dichloromethane (3 × 50 mL), and the organic phases were combined and dried over anhydrous sodium sulphate. The product was then concentrated under reduced pressure and filtered on a pad of silica gel using dichloromethane as eluent to give a yellow oil. The oil was diluted in dichloromethane (15 mL) and piperidine (30 eq) was added at 0 °C. The reaction mixture was stirred at room temperature for 3 h, acidified with hydrochloridric acid (10% solution), and extracted with dichloromethane (3 × 50 mL), and the organic phases were combined and dried over anhydrous sodium sulphate. The crude product was concentrated under reduced pressure and purified by flash chromatography using a 1:1 mixture of petroleum ether/ethyl acetate as eluent to give the pure ester.

*(E)-3,7-Dimethyloct-6-en-1-yl 3-(4-hydroxyphenyl)acrylate **10***: oil; 44%; [α]_D_^20^ 0.5 (c = 0.9, CHCl_3_); ^1^H-NMR δ 0.94 (d, *J* = 6.4 Hz, 3H), 1.22 (m, 1H), 1.38 (m, 1H), 1.51 (m, 1H), 1.60 (m, 1H), 1.61 (s, 3H), 1.67 (s, 3H), 1.76 (m, 1H), 1.99 (m, 2H), 4.26 (m, 2H), 5.11 (m, 1H), 6.28 (d, *J* = 16.0 Hz, 1H), 6.81 (m, Ar, 2H), 7.42 (m, Ar, 2H), 7.63 (d, *J* = 16.0 Hz, 1H); ^13^C-NMR δ 17.66, 19.43, 25.40, 25.73, 29.54, 35.48, 36.99, 63.40, 114.97, 115.99, 124.57, 126.63, 130.07, 131.37, 145.12, 158.51, 168.44; Anal. Calcd. for C_19_H_26_O_3_: C, 75.46; H, 8.67; Found: C, 75.44; H, 8.60.

*(E)-3,7-Dimethyloct-6-en-1-yl 3-(3,4-dihydrophenyl)acrylate****11***: brown solid; 47%; mp 100–101 °C; [α]_D_^20^ 2.9 (c = 0.4, CHCl_3_); ^1^H-NMR δ 0.93 (d, *J* = 6.4 Hz, 3H), 1.21 (m, 1H), 1.36 (m, 1H), 1.52 (m, 1H), 1.59 (m, 1H), 1.60 (s, 3H), 1.67 (s, 3H), 1.74 (m, 1H), 1.99 (m, 2H), 4.30 (m, 2H), 5.09 (m, 1H), 6.28 (d, *J* = 15.6 Hz, 1H), 6.88 (d, *J* = 8.4 Hz, Ar, 1H), 6.99 (dd, *J* = 2.1, 8.4 Hz, Ar, 1H), 7.12 (d, *J* = 2.1 Hz, Ar, 1H), 7.59 (d, *J* = 15.6 Hz, 1H); ^13^C-NMR δ 17.66, 19.42, 25.37, 25.72, 29.51, 35.42, 36.97, 63.61, 114.44, 115.14, 115.46, 122.46, 124.52, 127.17, 131.42, 144.03, 145.56, 146.78, 168.69; Anal. Calcd. for C_19_H_26_O_4_: C, 71.67; H, 8.23; Found: C, 71.60; H, 8.26.

*(E)-3,7-Dimethyloct-6-en-1-yl 3-(4-hydroxy-3-methoxyphenyl)acrylate **12***: oil; 37%; [α]_D_^20^ 0.6 (c = 0.4, CHCl_3_); ^1^H-NMR δ 0.93 (d, *J* = 6.8 Hz, 3H), 1.22 (m, 1H), 1.37 (m, 1H), 1.52 (m, 1H), 1.60 (m, 1H), 1.61 (s, 3H), 1.67 (s, 3H), 1.73 (m, 1H), 2.01 (m, 2H), 3.99 (s, 3H), 4.23 (m, 2H), 5.09 (m, 1H), 6.29 (d, *J* = 16.0 Hz, 1H), 6.89 (d, *J* = 8.4 Hz, Ar, 1H), 7.02 (d, *J* = 2.0 Hz, Ar, 1H), 7.06 (dd, *J* = 2.0, 8.4 Hz, Ar, 1H), 7.59 (d, *J* = 16.0 Hz, 1H); ^13^C-NMR δ 17.64, 19.44, 25.39, 25.71, 29.54, 35.55, 37.01, 55.90, 62.94, 109.32, 114.74, 115.61, 123.03, 124.59, 127.01, 131.31, 144.67, 146.78, 147.30, 168.41; Anal. Calcd. for C_20_H_28_O_4_: C, 72.26; H, 8.49; Found: C, 72.34; H, 8.40.

*(E)-3-Methylbut-2-en-1-yl 3-(4-hydroxyphenyl) acrylate **13***: oil; 45%; ^1^H-NMR δ 1.73 (s, 3H), 1.77 (s, 3H), 4.71 (d, *J* = 7.2 Hz, 2H), 5.41 (m, 1H), 6.29 (d, *J* = 16.0 Hz, 1H), 6.87 (m, Ar, 2H), 7.38 (m, Ar, 2H), 7.64 (d, *J* = 16.0 Hz, 1H); ^13^C-NMR δ 18.08, 25.82, 61.73, 114.91, 115.99, 118.42, 126.69, 130.07, 139.50, 145.19, 158.42, 168.36; Anal. Calcd. for C_14_H_16_O_3_: C, 72.39; H, 6.94; Found: C, 72.45; H, 6.96.

#### 4.1.3. General Procedure for the Synthesis of Compounds **14**–**17**

In a 30 mL glass pressure microwave tube, equipped with a magnetic stirrer bar, a few drops of concentrated sulphuric acid were added to a solution of hydroxycinnamic acid (*p*-coumaric acid or caffeic acid or ferulic acid) (1 eq) in methanol (for **14**–**16**) or ethanol (for **17**) (10 mL). The mixture was subjected to microwave irradiation (power: 150 W; temperature: 80 °C for **14**–**16** and 98 °C for **17**) for 15 min, basified with aqueous sodium bicarbonate (5% solution), and extracted with dichloromethane (3 × 5 mL). The collected organic phases were dried over anhydrous sodium sulphate and evaporated to dryness to give the pure ester.

*(E)-Methyl 3-(4-hydroxyphenyl)acrylate **14***: white solid; 82%; mp 125–127 °C ([58] 132–134 °C); ^1^H-NMR δ 3.79 (s, 3H), 5.37 (bs, 1H), 6.28 (d, *J* = 16.0 Hz, 1H), 6.85 (m, Ar, 2H), 7.42 (m, Ar, 2H), 7.63 (d, *J* = 16.0, 1H); ^13^C-NMR δ 51.75, 114.52, 115.91, 125.98, 129.89, 144.79, 158.12, 167.65. Anal. Calcd. for C_10_H_10_O_3_ C, 67.41; H, 5.66; Found: C, 67.53; H, 5.56.

*(E)-Methyl 3-(3,4-dihydroxyphenyl)acrylate **15***: brown solid; 88%; mp 155–156 °C ([59] 160 °C); ^1^H-NMR δ 3.80 (s, 3H), 6.25 (d, *J* = 16.0 Hz, 1H), 6.87 (d, *J* = 8.4 Hz, Ar, 1H), 7.01 (dd, *J* = 2.0, 8.4 Hz, Ar, 1H), 7.07 (d, *J* = 2.0 Hz, Ar, 1H), 7.58 (d, *J* = 16.0 Hz, 1H); ^13^C-NMR δ 51.78, 114.33, 115.27, 115.49, 122.46, 127.52, 143.74, 145.03, 146.28, 168.18. Anal. Calcd. for C_10_H_10_O_4_; C, 61.85; H, 5.19; Found: C, 62.05; H, 5.78.

*(E)-Methyl 3-(4-hydroxy-3-methoxyphenyl)acrylate **16***: brown solid; 92%; mp 62–64 °C ([60] 65 °C); ^1^H-NMR δ 3.75 (s, 3H), 3.83 (s, 3H), 6.14 (bs, 1H), 6.25 (d, *J* = 16.0 Hz, 1H), 6.86 (d, *J* = 8.0 Hz, Ar, 1H), 6.96 (d, *J* = 2.0 Hz, Ar, 1H), 6.96 (dd, *J* = 2.0, 8.0 Hz, Ar, 1H), 7.58 (d, *J* = 16.0 Hz, 1H); ^13^C-NMR δ 51.64, 55.84, 109.52, 114.72, 114.81, 122.98, 126.78, 145.12, 146.92, 148.12, 167.92. Anal. Calcd. for C_11_H_12_O_4_ C, 63.45; H, 5.81; Found: C, 63.51; H, 5.76.

*(E)-Ethyl 3-(4-hydroxyphenyl)acrylate **17**:* brown solid; 87%; mp 70–72 °C ([61] 73–74 °C); ^1^H-NMR δ 1.32 (t, *J =* 7.1 Hz, 3H), 4.26 (q, *J* = 7.1 Hz, 2H), 6.39 (d, *J* = 16.0 Hz, 1H), 7.35 (m, Ar, 2H), 7.47 (m, Ar, 2H), 7.58 (d, *J* = 16.0 Hz, 1H); ^13^C-NMR δ 14.43, 60.74, 119.14, 124.58, 129.54, 132.22, 133.52, 143.29, 166.78. Anal. Calcd. for C_11_H_12_O_3_: C, 68.74; H, 6.29; Found: C, 68.81; H, 6.34.

#### 4.1.4. Procedure for the Synthesis of Compound **13** with Lipase

To a solution of **17** (1 eq) in cyclohexane (2.5 mL), 3,3-dimethylallyl alcohol (2 eq) was added. The reaction mixture was stirred at 60 °C for 15 min at a speed of 300 rpm. The reaction was initiated by adding a known fixed quantity of lipase (100 mg). The progress of the reaction was monitored by TLC using a 1:1 mixture of petroleum ether/ethyl acetate as eluent. After three days, the starting material was still present and another aliquot of lipase (100 mg) was added, and the mixture was left stirring at 60 °C for two additional days. The reaction mixture was filtered over Buchner funnel, solvent concentrated under reduced pressure, and purified by flash chromatography using a 1:1 mixture of petroleum ether/ethyl acetate as eluent to obtain compound **13** (0.19 g, 80% yield).

#### 4.1.5. General Procedure for the Synthesis of Compounds **4**, **6, 7**, and **9**

Compound **14** or **15** or **16** (1 eq) was dissolved in dry acetone (15 mL) and then anhydrous potassium carbonate (1 eq) and appropriated alkenyl bromide (1 eq) were added. The resulting mixture was stirred at 50 °C for 12 h, then sodium hydroxide 2 N (15 mL) was added and the reaction mixture was stirred at 90 °C for an additional 3 h. The cooled solution was acidified to pH 2 with hydrochloridric acid (10% solution) and extracted with dichloromethane (3 × 50 mL). The collected organic phases were dried over anhydrous sodium sulphate and evaporated to dryness to give the pure ether.

*(E)-3-(4-(Allyloxy)phenyl)acrylic acid **4***: white solid; 85%; mp 161–162 °C ([62] 160 °C); ^1^H-NMR δ 4.56 (d, *J* = 5.2 Hz, 2H), 5.29–5.44 (series of m, 2H), 6.03 (m, 1H), 6.29 (d, *J* = 16.0 Hz, 1H), 6.91 (m, Ar, 2H), 7.49 (m, Ar, 2H), 7.73 (d, *J* = 16 Hz, 1H); ^13^C-NMR δ 68.85, 114.72, 115.13, 118.08, 126.91, 130.07, 132.66, 146.67, 160.73, 172.55. Anal. Calcd. for C_12_H_12_O_3_ C, 70.57; H, 5.92; Found C, 70.78; H, 5.87.

*(E)-3-(4-(Allyloxy)-3-methoxyphenyl)acrylic acid **6***: white solid; 53%; mp 152–154 °C ([63] 151–153 °C); ^1^H-NMR δ 3.91 (s, 3H), 4.66 (m, 2H), 5.30–5.44 (series of m, 2H), 6.08 (m, 1H), 6.29 (d, *J* = 16.0 Hz, 1H); 6.86 (d, *J* = 8.4 Hz, Ar, 1H), 7.08 (dd, *J* = 2.0, 8.4 Hz, Ar, 1H), 7.09 (d, *J* = 2.0 Hz, Ar, 1H), 7.71 (d, *J* = 16.0 Hz, 1H); ^13^C-NMR δ 55.92, 69.70, 110.08, 112.71, 114.90, 118.51, 122.92, 127.14, 132.61, 147.01, 149.52, 150.46, 172.72. Anal. Calcd. for C_13_H_14_O_4_ C, 66.66; H, 6.02; Found: C, 66.87; H, 6.12.

*(E)-3-(4-((3-Methylbut-2-en-1-yl)oxy)phenyl)acrylic acid **7***: white solid; 69%; mp 146–147 °C ([16] 148–150 °C); ^1^H-NMR δ 1.73 (s, 3H), 1.79 (s, 3H), 4.52 (d, *J* = 6.4 Hz, 2H), 5.47 (s, 1H), 6.28 (d, *J* = 16 Hz, 1H), 6.89 (d, *J* = 8.8 Hz, Ar, 2H), 7.47 (d, *J* = 8.4 Hz, Ar, 2H), 7.73 (d, *J* = 16 Hz, 1H); ^13^C-NMR δ 25.81, 29.19, 64.91, 114.62, 115.04, 119.11, 126.67, 130.04, 138.73, 146.60, 161.04, 171.36. Anal. Calcd. for C_14_H_16_O_3_ C, 72.39; H, 6.94; Found C, 72.59; H, 6.03.

*(E)-3-(4-(((E)-3,7-Dimethylocta-2,6-dien-1-yl)oxy)-3-methoxyphenyl)acrylic acid **9**:* white solid; 75%; mp 59–60 °C ([64] 60–61 °C); ^1^H-NMR δ 1.63 (s, 3H), 1.70 (s, 3H), 1.77 (s, 3H), 2.01–2.24 (series of m, 4H), 3.95 (s, 3H), 4.62 (m, 2H), 5.11 (m, 1H), 5.44 (m, 1H), 6.33 (d, *J* = 16.0 Hz, 1H), 6.86 (d, *J* = 7.6 Hz, Ar, 1H), 7.01–7.15 (series of m, Ar, 2H), 7.37 (d, *J* = 16.0 Hz, 1H); ^13^C-NMR δ 16.70, 17.70, 25.70, 26.20, 39.53, 55.91, 65.82, 109.91, 112.52, 114.71, 119.11, 123.01, 123.71, 126.81, 131.84, 141.22, 147.01, 149.52, 150.83, 172.11. Anal. Calcd. for C_20_H_26_O_4_ C, 72.70; H, 7.93; Found: C, 72.80; H, 7.92.

#### 4.1.6. Synthesis of Compound **5**

*(E)-3-(4-(Allyloxy)-3-hydroxyphenyl)acrylic acid **5**:* Compound **15** (0.5 g, 2.57 mmol) was dissolved in dry acetone (15 mL) and then anhydrous potassium carbonate (0.35 g, 2.57 mmol) and allyl bromide (0.31 g, 2.57 mmol) were added. The resulting mixture was stirred at 50 °C for 12 h. The cooled solution was acidified to pH 2 with hydrochloridric acid (10% solution) and extracted with dichloromethane (3 × 50 mL). The collected organic phases were dried over anhydrous sodium sulphate and evaporated. The crude product was purified by flash chromatography using a mixture of 3:1 petroleum ether/acetone as eluent to give compound **18** as a white solid (0.47 g, 78%).

*(E)-Methyl 3-(4-(allyloxy)-3-hydroxyphenyl)acrylate **18***: mp 95–96 °C ([65] 94–95 °C); ^1^H-NMR δ 3.78 (s, 3H), 4.65 (d, *J* = 5.6 Hz, 2H), 5.33–5.43 (series of m, 2H), 5.69 (s, 1H), 6.08 m, 1H), 6.30 (d, *J* = 15.6 Hz, 1H), 6.83 (d, *J* = 8.4 Hz, Ar, 1H), 6.99 (dd, *J* = 2.0 8.4 Hz, Ar, 1H), 7.14 (d, *J* = 2.0 Hz, Ar, 1H), 7.61 (d, *J* = 15.6 Hz, 1H); ^13^C-NMR δ 51.59, 69.80, 11.84, 113.21, 115.94, 118.80, 121.64, 123.13, 128.20, 132.26, 144.60, 146.02, 147.39, 167.68. Anal. Calcd. for C_14_H_16_O_4_ C, 67.73; H, 6.50; Found: C, 67.52; H, 6.42. To compound **18** (0.47 g, 2.00 mmol) in a 3:1 solution of MeOH:H_2_O, sodium hydroxide 2 N (15 mL) was added and the mixture was stirred at 90 °C for 3 h. The cooled solution was acidified to pH 2 with hydrochloridric acid (10% solution) and extracted with dichloromethane (3 × 50 mL). The collected organic phases were dried over anhydrous sodium sulphate and evaporated to dryness to give the pure **5** as white solid (0.33 g, 75%); mp 184–185 °C; ^1^H-NMR δ (acetone d_6_) 4.66 (m, 2H), 5.26 (m, 1H), 5.46 (m, 1H), 6.07 (m, 1H), 6.32 (d, *J* = 16 Hz, 1H), 7.01 (d, *J* = 8.4 Hz, Ar, 1H), 7.09 (dd, *J* = 2.4, 8.4 Hz, Ar, 1H), 7.19 (d, *J* = 2.4, Hz, Ar, 1H), 7.56 (d, *J* = 16 Hz, 1H); ^13^C-NMR δ 64.41, 112.87, 113.95, 115.94, 117.05, 121.08, 128.03, 133.44, 144.61, 147.08, 148.52, 167.09. Anal. Calcd. for C_12_H_12_O_4_ C, 65.45; H, 5.49; Found: C, 65.52; H, 5.32.

### 4.2. Fungal Strains and Culture

Two monosporic isolates collected from human samples from an Italian hospital (Table 2) were selected as representative of each *Fusarium oxysporum*, *F. solani*, and *F. fujikuroi* species complexes. Conidial suspensions of each strain were pre-cultured in a carboxymethyl cellulose medium (CMC; [66]) for 5 days on a rotary shaker at 24 °C and 180 rpm. Cultures were filtered through four layers of sterile cheesecloth, and spores were collected by centrifugation, adjusted to 1 × 10^6^ colony-forming units (CFU)/mL in sterile water, and used as inoculum.

### 4.3. Evaluation of the Antifungal Activity of Compounds ***1**–**13*** in FMM Solid Medium

A total of 13 phenolic compounds (Table 1) were tested for their antifungal activity against the six *Fusarium* spp. isolates (Table 2) in Fusarium minimal medium (FMM) [67]. Each phenolic compound was resuspended in H_2_O/gellan 0.1 % solution and sonicated at room temperature for 1 h at 80 Hz (Elmasonic P 180 H, Elma Schmidbauer GmbH, Germany). Solid FMM with nitrate sodium NaNO_3_ as nitrogen source was distributed into Ø90 mm Petri dishes (15 mL/Petri dish) and amended with each compound at a final concentration of 0.5 mM at a temperature of 45 °C. Ten microliters of the conidial suspension of each strain were spotted onto the center of the Petri dish amended FMM. Antifungal activity of each compound was measured after 5 d of growth at 25 °C in the dark and expressed as the colony diameter (percentage relative to control). Three replicates were prepared for each isolate/inhibitor combination and the experiment was repeated once.

### 4.4. Evaluation of the Antifungal Activity of p-Coumaric Acid 3,3′-Dimethyl Allyl Ester (***13***) in FMM Liquid Medium

The conventional antifungal drugs used in the study were AmB and TRB. AmB was purchased by Sigma Aldrich (A2942, Germany) as a standard solution. TRB was extracted from Terbinafine Hexal 250 mg tablets by fine crushing and dissolution in a solution 1:2 (v:v) dichloromethane and water. The emulsion was stirred at room temperature until two phases clearly appeared. The organic phase was extracted and dried on Na_2_SO_4_ and the TRB was recovered in neat form after evaporation of the solvent under vacuum. NMR spectra of the solid extract confirmed the presence of TRB with a purity ≅98%. TRB was dissolved in 60% ethanol/H_2_O (*v*/*v*), while AmB was diluted with water to reach the desired concentration and was frozen in aliquots at −20 °C. AmB and TRB concentrations were selected according to clinical dosage and standard experimental procedures with some modifications [68].

The minimal inhibitory concentration (MIC) and the lethal dose 50 (LD_50_) of each strain were assayed by a standardized micro-dilution method in the 96-well plate. Two-fold serial dilutions of each antifungal agent in liquid FMM in a total volume of 200 μL were tested. Further, 10 μL of fungal spore suspension (4 × 10^6^ CFU/mL) was added. A blank control with water was used for each treatment. The optical density mOD of each microplate well was measured at 2 h intervals during 72 h of incubation with a microplate spectrophotometer SpectrostarNano (Euroclone, Germany) at a 595 nm wavelength. The inhibitory activity of each compound was expressed as MIC, representing the lowest concentration of active ingredient (μM) that is sufficient to inhibit the absorbance signal, whereas the LD_50_ of each compound was calculated as the concentration of active ingredient (μM) able to reduce by 50% the mOD_595_ signal. The experiments were repeated at least two times in quadruplicate.

### 4.5. Optical Microscopy Examination

A drop (15 μL) of the total volume present on the wells corresponding to the MIC of each isolate/compound combination was pipetted after 72 h of incubation onto a glass slide. A clean glass cover slip was placed on the sample prepared with emulsion oil. Each slide was examined at 100× for the presence of mycelium, branched hyphae, fialides, microconidia, and germinating spores, using an optical microscope (LEICA ICC50) at a scale of 20 µm.

### 4.6. Data Acquisition and Analysis

In the first screening, an analysis of variance (one-way ANOVA) followed by multiple comparisons by Tukey HSD test at the significance level *p* < 0.05 using Minitab for Windows, release 17 was performed.

In the second screening, data were recorded and analysed with Mars Data Analysis Software, BMG Labtech, and exported to Microsoft Excel for generation of the graphs. Graphs for the determination of the MIC and LD_50_ (Figures S4–S9) were generated for an incubation time between 0 h and 48 h because, after this interval time, the drug free-test (control) curve started to reach the stationary phase for almost all strains investigated. Optical microscopy images were captured and treated with LAS V4.13 Leica application software.

## 5. Conclusions

The design of cinnamic derivatives **4**–**13** was focused on both electronic and steric modification of the parent compounds **1**–**3** by esterification and etherification reaction with bioactive prenylated chains, with the aim to enhance the antifungal activity of the final compound. Compounds **1**–**3** are commercially available at a reasonable price and offer a successful example of repositioning of natural compounds. In this study, we provided data that may contribute to increasing the knowledge about the promotion of the importance of antifungal susceptibility testing. *p*-Coumaric acid 3,3′-dimethyl allyl ester **13**, a component of propolis, showed good antifungal activities against *Fusarium* spp., causing onychomycosis, and identifies prenylated hydroxy cinnamic acids as interesting pharmacophore for developing new drugs effective against this pathology. The activity of this compound will be investigated over a larger number of isolates belonging to different species complexes and haplotypes. Furthermore, the mechanism of action of compound **13** needs to be fully characterized and possibly tested in combination with other bioactive molecules that may be enabled to reach their target within the fungal cell.

Noteworthy, this study cannot be adopted as a clinical guideline, and the MIC values obtained must be tested in an appropriately designed clinical study.

## Figures and Tables

**Table 1:** Values of the logarithm of LogP of compounds **1**–**13** for *n*-octanol/water, estimated by ChemBioDraw Ultra 13.

Compound	LogP	Compounds	LogP	Compounds	LogP
**1**	1.15	**6**	2.37	**11**	4.51
**2**	1.42	**7**	3.04	**12**	4.78
**3**	1.54	**8**	2.11	**13**	3.04
**4**	2.50	**9**	4.44		
**5**	2.11	**10**	4.90		

**Table 2:** List of *Fusarium* spp. isolates tested in this study.

Species/Species Complex/Sequence Type (ST)	NRRL n.^a^	PVS-Fu n.^b^	Diagnosis	Isolate Source	Date
*F. oxysporum/*FOSC/ST33	46603	89	Onychomycosis	Toe	2004
*F. oxysporum/*FOSC/ST33	46606	91	Onychomycosis	Toe	2005
*F. keratoplasticum*/FSSC/ST2bb	46443	93	Dermatomycoses	Foot	2004
*F. solani*/FSSC/ST5aa	44903	96	Onychomycosis	Toe	2006
*F. verticillioides*/FFSC	46599	87	Onychomycosis	Toe	2007
*F. verticillioides*/FFSC	46442	115	Onychomycosis	Toe	2005

**^a^** NRRL n. Collection number of Agricultural Research Service (ARS); **^b^** PVS-Fu n. Collection number of Dipartimento di Agraria, Sezione Patologia Vegetale ed Entomologia, Sassari, Italy.

**Table 3:** In vitro susceptibility of strains belonging to the three *Fusarium* species complexes, isolated from onychomycosis and dermatomycosis against the selected compound *p*-coumaric acid 3,3′-dimethyl allyl ester **13** and the two conventional drugs TRB **^b^** and AmB **^c^**.

Species/Species Complex/Sequence Type (ST)	^a^ PVS-Fu n.	Ester 13		^b^ TRB		^c^ AmB	
		^d^ MIC (µM)	^e^ LD_50_ (µM)	MIC (µM)	LD_50_ (µM)	MIC (µM)	LD_50_ (µM)
*F. oxysporum*/FOSC/(ST33)	89	>125–250	31–62	>256	8–16	>135	16.8–33.7
*F. oxysporum*/FOSC/(ST33)	91	>125–250	62–125	>256	16–64	>135	33.7–67.5
*F. keratoplasticum*/FSSC/(ST2bb)	93	62	<7.8	128–256	2.0–4.0	33.7–67.5	>2.1–4.2
*F. solani*/FSSC/(ST5aa)	96	62–125	<7.8	64–128	2.0–4.0	4.2–2.1	1.0
*F. verticillioides*/FFSC	87	500	62–125	>256	2.0–4.0	>135	2.1–4.2
*F. verticillioides*/FFSC	115	125–250	<7.8	>256	2.0–4.0	>135	8.4–16.8

**^a^** PVS-Fu n. Collection number of Dipartimento di Agraria, Sezione Patologia Vegetale ed Entomologia, Sassari, Italy; **^b^** TRB: terbinafine; **^c^** AmB: amphotericin B; **^d^** minimal inhibitory concentration (MIC) range determined visually according to mOD absorbance values (nm) detected after 48 h of incubation at 25 °C; **^e^** lethal dose 50 (LD_50_) range determined visually according to mOD absorbance values (nm) detected after 48 h of incubation at 25 °C.

**Table 4:** Optical microscopy (100×) images of the fungal growth of *Fusarium* spp. after 72 h on liquid Fusarium minimal medium (FMM) amended with 250, 256, or 135 μM of *p*-coumaric acid 3,3′-dimethyl allyl ester **13**, terbinafine (TRB), or amphotericin B (AmB), respectively, in comparison with the untreated control.

Species	Control	Ester 13	TRB	AmB
	0 (µM)	MIC (250 µM)	MIC (256 µM)	MIC (135 µM)
***F. keratoplasticum***	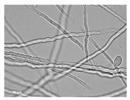	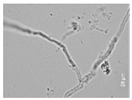	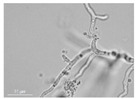	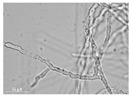
	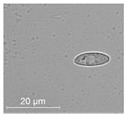	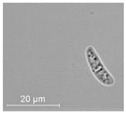	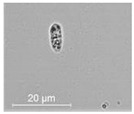	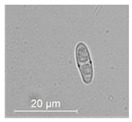
***F. solani***	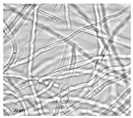	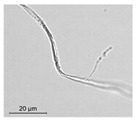	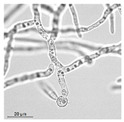	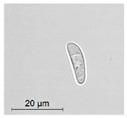
	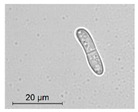	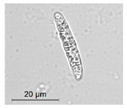	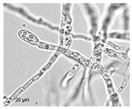	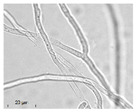
***F. oxysporum***	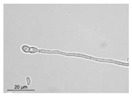	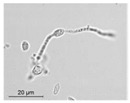	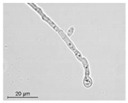	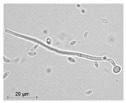
	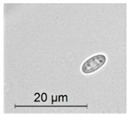	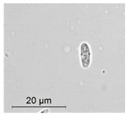	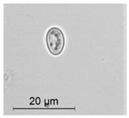	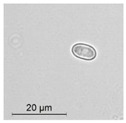
***F. verticillioides***	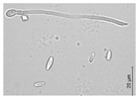	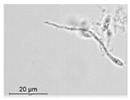	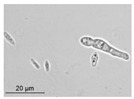	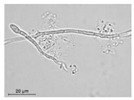
	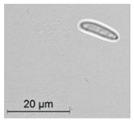	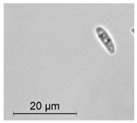	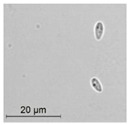	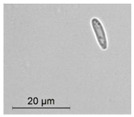

## Supplementary Materials

The following are available online. Figures S1–S3: Antifungal activity of compounds **1**–**13** against FOSC, FFSC, and FFSC, respectively. Figures S4–S9: MIC and LD_50_ ranges expressed as absorbance (milliOD) at 595 nm at 48 h of ester **13** (A), TRB (B), and AmB (C) against FOSC, FSSC, and FFSC strains, respectively.

## Sample Availability

Samples of the compounds **1**–**13** are available from the authors.
