# Supplementary material for: Prenylated Trans-Cinnamic Esters and Ethers against Clinical Fusarium spp.: Repositioning of Natural Compounds in Antimicrobial Discovery
Source: Molecules. 2021 Jan 27;26(3):658. doi: 10.3390/molecules26030658 (PMC7865625; doi:10.3390/molecules26030658)
Supplement: Supplementary file 1 [file molecules-26-00658-s001.pdf]

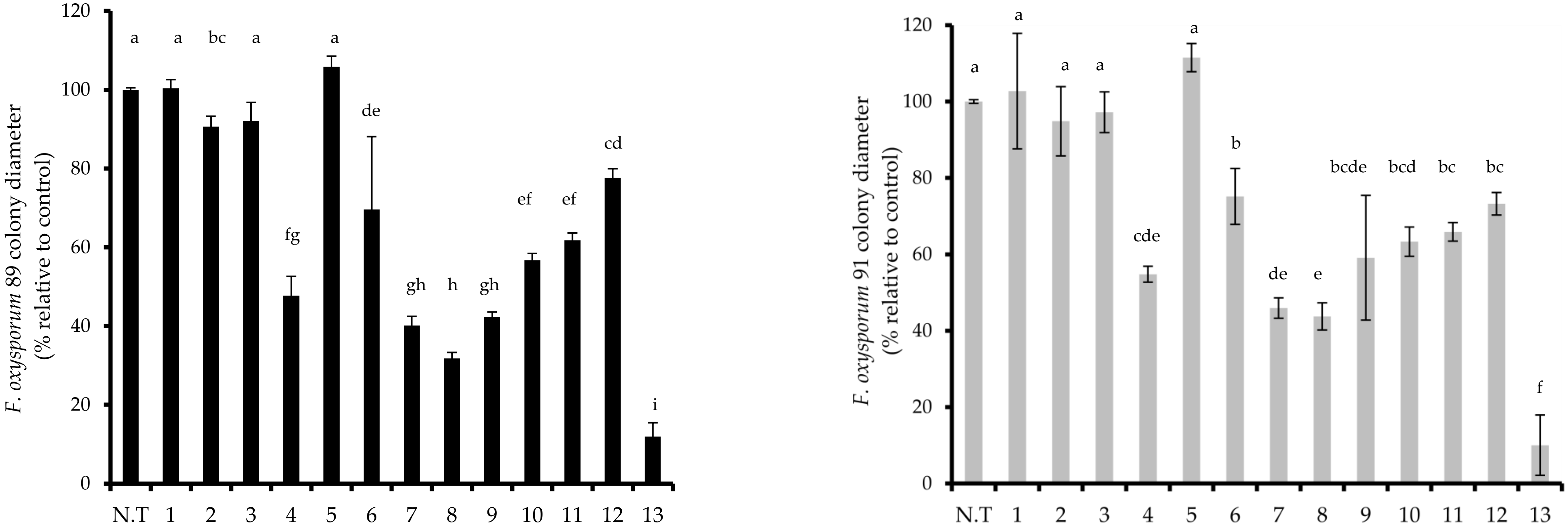

Figure S1. Antifungal activity of compounds **1-13** against FOSC.

*N.T: Not treated (drug-free test).
Compounds that id not share the same letter are statistically different at $P < 0.05$.

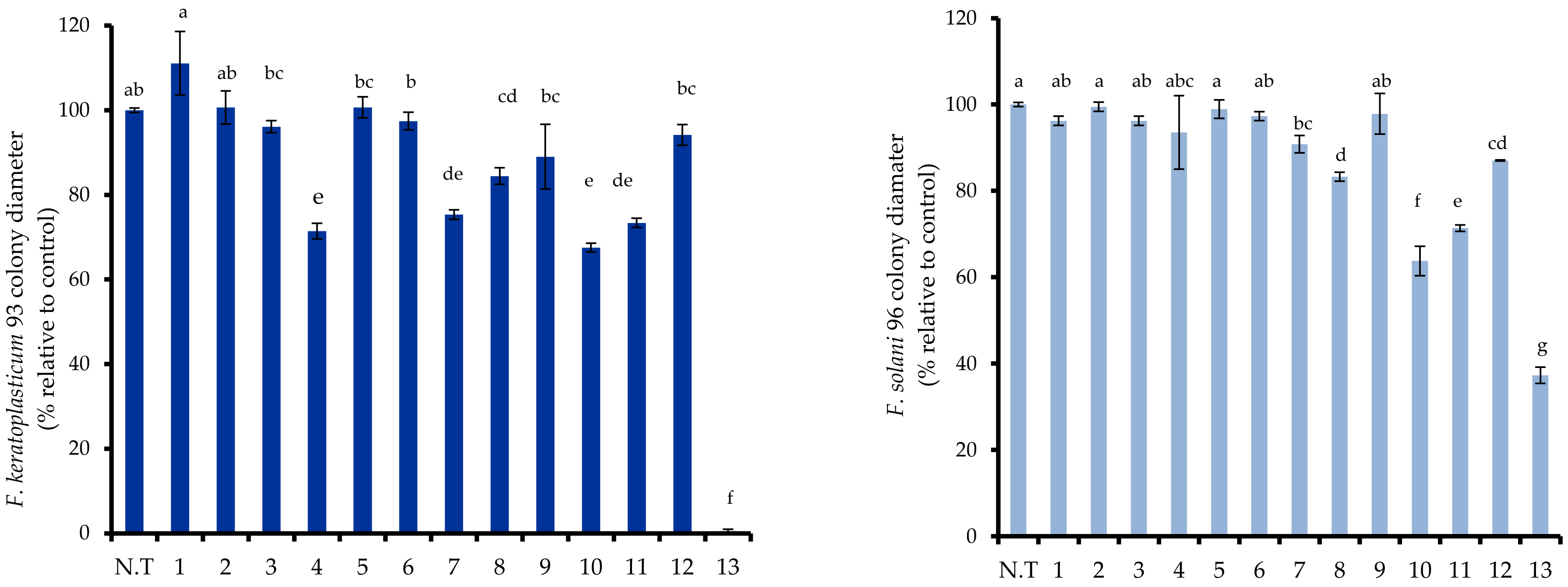

Figure S2. Antifungal activity of compounds **1-13** against FSSC.

*N.T: Not treated (drug-free test).

Compounds that id not share the same letter are statistically different at $P < 0.05$.

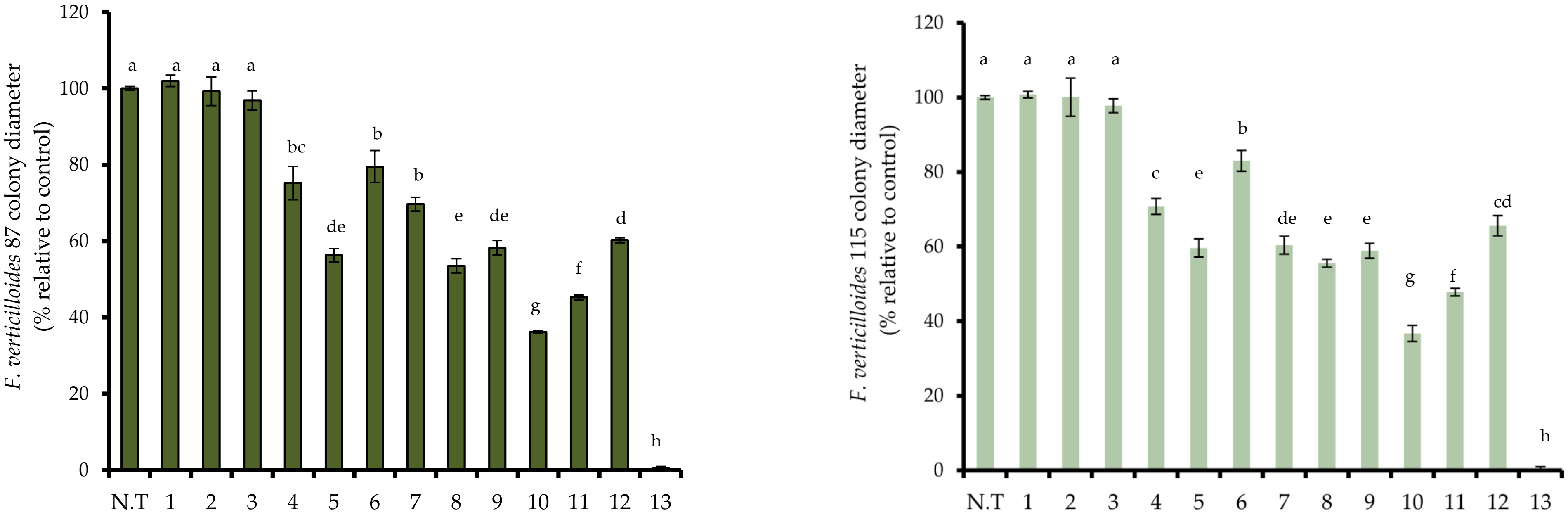

Figure S3. Antifungal activity of compounds **1**-**13** against FFSC.

*N.T: Not treated (drug-free test).

Compounds that id not share the same letter are statistically different at $P < 0.05$.

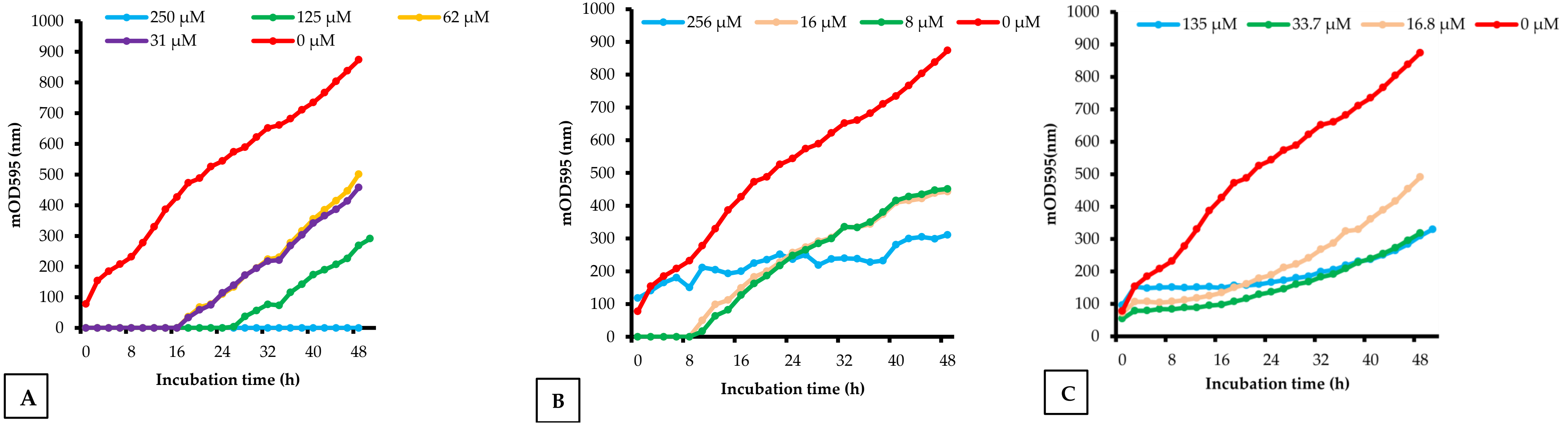

Figure S4. MIC and LD_{50} ranges expressed as absorbance (milliOD) at 595 nm at 48 h of Ester **13** (A), TRB (B) and AmB (C) against *F. oxysporum* 89.

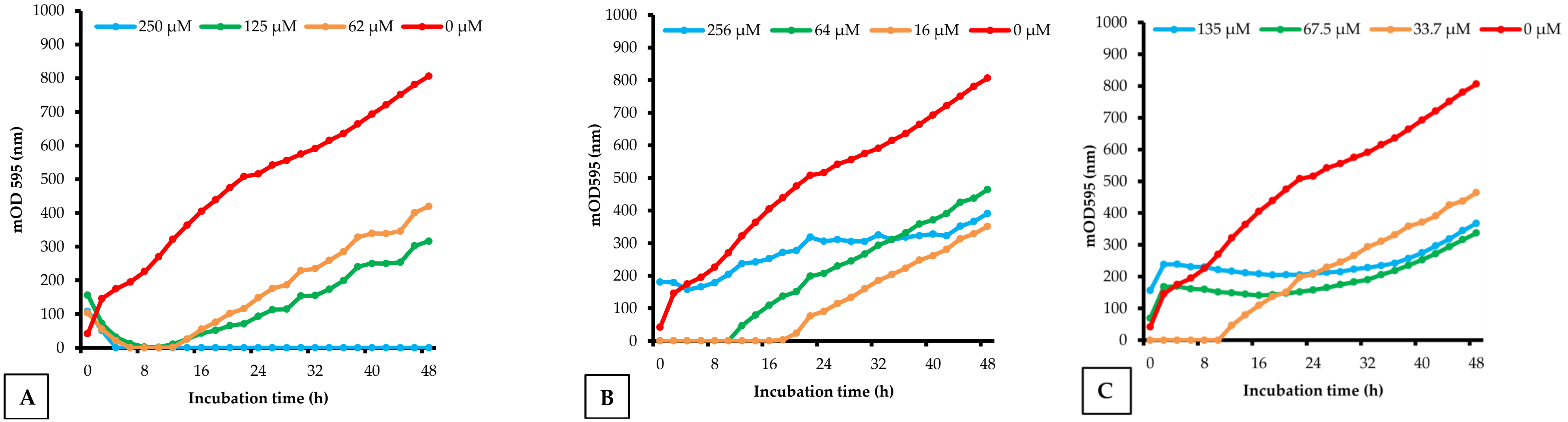

Figure S5. MIC and LD_{50} ranges expressed as absorbance (milliOD) at 595 nm at 48 h of Ester **13** (A), TRB (B) and AmB (C) against *F. oxysporum* 91.

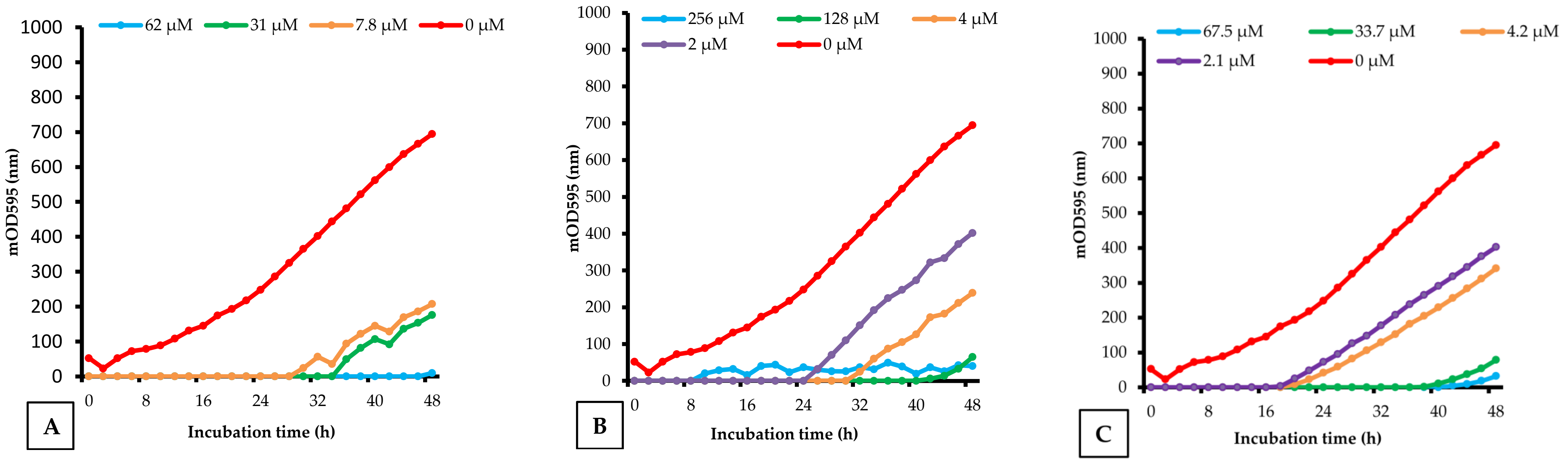

Figure S6. MIC and LD50 ranges expressed as absorbance (milliOD) at 595 nm at 48 h of Ester **13** (A), TRB (B) and AmB (C) against *F. keratoplasticum* 93.

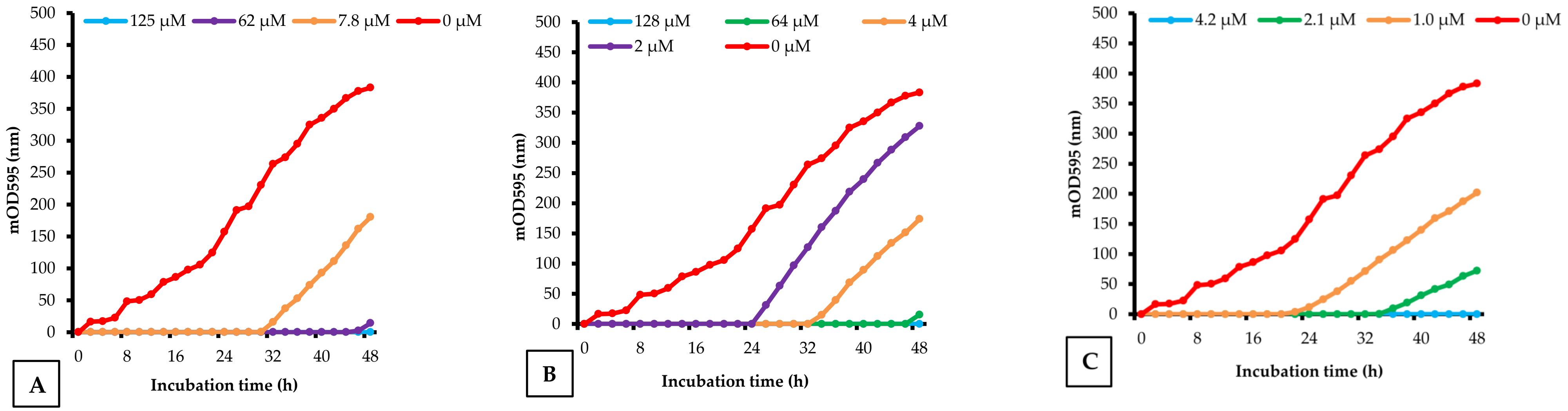

Figure S7. MIC and LD_{50} ranges expressed as absorbance (milliOD) at 595 nm at 48 h of Ester **13** (A), TRB (B) and AmB (C) against *F. solani* 96.

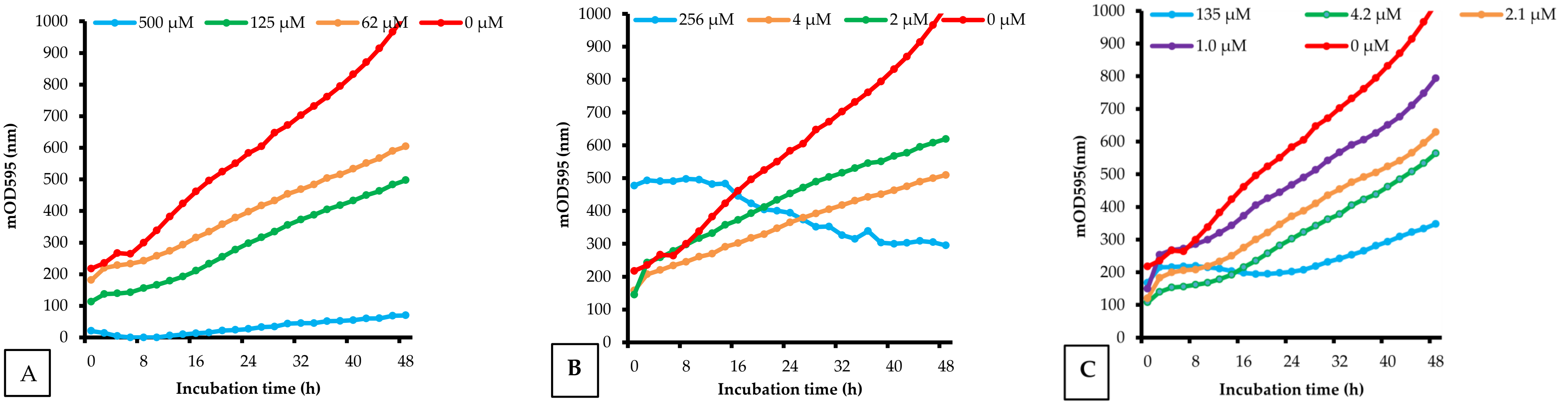

Figure S8. MIC and LD_{50} ranges expressed as absorbance (milliOD) at 595 nm at 48 h of Ester **13** (A), TRB (B) and AmB (C) against *F. verticillioides* 87.

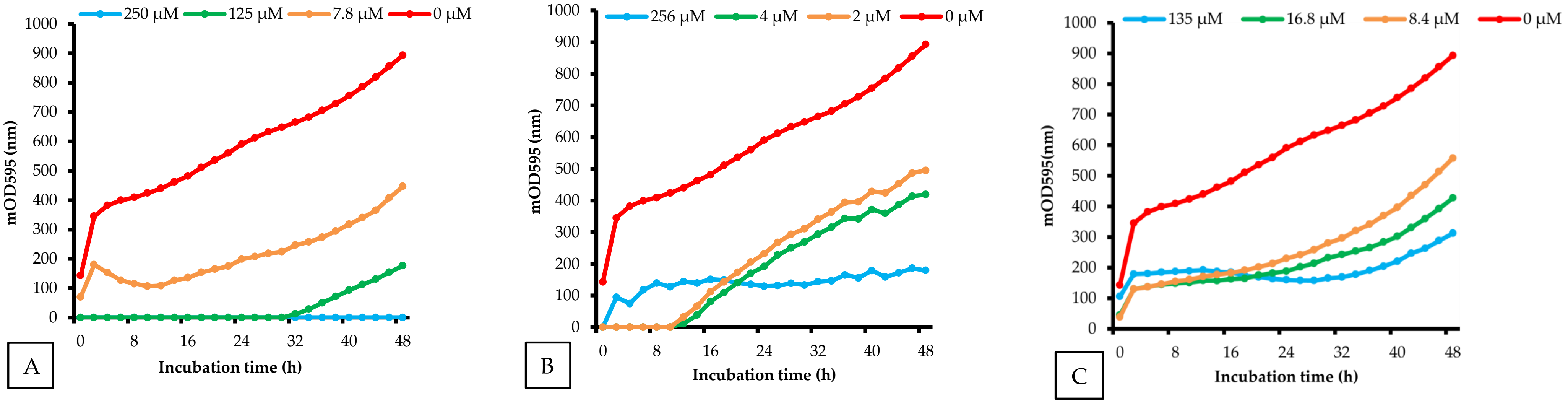

Figure S9. MIC and LD$_{50}$ ranges expressed as absorbance (milliOD) at 595 nm at 48 h of Ester **13** (A), TRB (B) and AmB (C) against *F. verticillioides* 115.
